# A Cross-European Study of Informal Carers’ Needs in the Context of Caring for Older People, and their Experiences with Professionals Working in Integrated Care Settings

**DOI:** 10.5334/ijic.5547

**Published:** 2021-07-08

**Authors:** Eliva Atieno Ambugo, Simone R. de Bruin, Lina Masana, Julie MacInnes, Nuri Cayuelas Mateu, Terje P. Hagen, Borja Arrue

**Affiliations:** 1 Department of Health, Social and Welfare Studies - University of South-Eastern Norway, Norway; 2 Department of Health Management and Health Economics, Institute of Health and Society, University of Oslo, Oslo, Norway; 3 National Institute for Public Health and the Environment, Center for Nutrition, Prevention and Health Services, Bilthoven, The Netherlands; 4 Agency for Health Quality and Assessment of Catalonia (AQuAS), Catalan Government Department of Health, Barcelona, Spain; 5 Centre for Health Services Studies, University of Kent, Canterbury, UK; 6 AGE Platform Europe, Brussels, Belgium

**Keywords:** informal carers, informal caregivers, person-centredness, needs assessment, goal setting

## Abstract

**Introduction:**

Informal carers are increasingly relied on for support by older people and the health and social care systems that serve them. It is therefore important that health and social care professionals are knowledgeable about and responsive to informal carers’ needs. This study explores informal carers’ own needs within the context of caregiving; and examines, from the informal carers’ perspective, the extent to which professionals assess, understand and are responsive to informal carers’ needs.

**Methods:**

We interviewed (2016–2018) 47 informal carers of older people being served by 12 integrated care initiatives across seven countries in Europe. The interviews were thematically coded inductively and analysed.

**Results:**

Informal carers reported that professionals treated them with respect and made efforts to assess and respond to their needs. However, even though professionals encouraged informal carers to look after themselves, informal carers’ needs (e.g., for respite, healthcare) were insufficiently addressed, and informal carers tended to prioritize older people’s needs over their own.

**Discussion and conclusion:**

Informal carers need better support in caring for their own health. Health professionals should have regular contact with informal carers and proactively engage them in ongoing needs assessment, setting action plans for addressing their needs, and identifying/accessing appropriate support services. This will be important if informal carers are to continue their caregiving role without adverse effects to themselves.

## Introduction

Many older people who live at home receive care (e.g., support with activities of daily living) from informal carers, who include relatives, neighbours and friends [1,2,3]. While informal carers have reported some positive effects of caregiving (e.g., satisfaction of caring for a loved one) [3,4], caregiving is also known to exert some negative effects on informal carers’ health and wellbeing (e.g., exposure to stress) [3,5,6]. Given the key role that informal carers play in the delivery of care to older people, thereby supporting them to live at home longer and avoid costly institutional care, there is a need for health and social care professionals (henceforth professionals) to work more closely with informal carers to identify and support them in meeting their health and social care needs [1,3]. The current study explores informal carers’ own needs within the context of caregiving, and their experiences with professionals working within integrated health and social care systems in Europe. The findings will help identify strengths and deficits in the ways in which health and social care systems ‘care for informal carers’, and in so doing generate knowledge that can inform future efforts aimed at better supporting informal carers.

## Background

It is estimated that around 80% of all care received by people of all ages in the EU is provided by informal carers, of whom two thirds are women [7]; and 7% and 9% of adults ages 35–49 and 50–64 respectively reported that they provide care to an older person on daily basis [8]. Informal care has been estimated to have an economic value equivalent to 50–90% of the overall cost of long-term care across the EU [9]. In the context of demographic ageing and the subsequent increase in the number of older people in need of care, informal care is seen as a way of containing the costs associated with formal care delivery in many countries [9].

Although informal carers report positive experiences from caregiving, including a sense of giving back to someone who has cared for them, and the satisfaction of knowing that their loved one is getting excellent care [4], caregiving may also negatively affect the health and wellbeing of informal carers. Many informal carers are themselves older in age (e.g., spouses of older people) and may face a greater risk of experiencing carer burden due to their own health and social care needs [10,11]. Caring for sick or older loved ones often comes with demands that can lead to challenges, for example: poor diet and limited exercise [10]; reduced quality of life, stress, anxiety and depression [5,12]; social isolation due to being or feeling unable to leave the home because of caring responsibilities [6]; and reduced income [5,13]. Approximately 5% and 8% of midlife women who provide care in the Scandinavian countries and Western Europe reduce their paid work hours or leave the labour force due to caring responsibilities [14].

Informal carers have varied needs that can change over time [11,15]. Health and social care professionals can play an important role in assessing carers’ needs, and in referring them to or providing them with support in undertaking their role. Such support can enable informal carers to take better care of their own health and wellbeing [1], which in turn may facilitate carers’ important contributions to the sustainability of long-term care. However, there are barriers to professionals paying closer attention to informal carers’ wellbeing, including lack of clear mandates/requirements to do so, and professionals’ busy schedules and limited experience or skills in working with informal carers [1,3]. In the United Kingdom (UK), it is estimated that 70% of informal carers come into contact with professionals due to their own health needs, or because of the needs of the person they are caring for. Yet only one in ten informal carers is identified as a carer by professionals [1]. Moreover, many informal carers do not recognise themselves as having a caring role. Rather they identify themselves as a partner in an ongoing, reciprocal relationship [1]. Informal carers do however consider professionals to be a source of information and support when these professionals are known to the informal carers.

Given 1) a health and social care landscape that is primarily centred around older people compared to their informal carers [3], 2) the crucial role that informal carers play in caring for older people, and 3) informal carers’ own health and social care needs that also warrant attention [3,16], the aim of this study is to examine, from the informal carers’ perspective, the extent to which professionals assess, understand and are responsive to informal carers’ needs. Integrated care is seen as a promising approach to providing care for older people in their home environments in a proactive and coordinated manner, centered around theirs’ and their informal carers’ needs [17]. Person-centered care is an important component of integrated care [17,18]. Its key elements include: promoting the client’s active participation in making decisions about and managing his/her health and social care needs, promoting a cooperative relationship with the client (respect, active listening, good communication), understanding the client’s specific needs and concerns (his/her preferences, priorities), addressing the client’s varied needs (paying attention to the whole person), providing coordinated care [18,19]. We aim to gain an understanding (based on informal carers’ own reports) of how professionals in such settings consider and respond to informal carers’ own needs, and the extent to which those efforts are person-centered.

## Methods

### Study design

This study was part of the SUSTAIN (Sustainable Tailored Integrated Care for older people in Europe) project—April 2015 and March 2019. Employing a multiple embedded case study design [20,21], data was collected from thirteen established integrated care initiatives for older people across Europe: Austria, Estonia, Germany, Norway, Spain, the Netherlands and the UK. The SUSTAIN project is described in further detail elsewhere [22], and was funded under Horizon 2020 – the Framework Programme for Research and Innovation (2014–2020) from the European Commission.

Different types of support services (Appendix-A), organized at the national/regional levels and by voluntary organizations, were available for informal carers in this study; and the health and social care organizations involved in SUSTAIN mainly provided informal carers with information about- or referred carers to the services. All the countries have universal coverage for primary and tertiary healthcare. Germany and Norway also have universal coverage for long-term care and social services; whereas the other countries have mixed-coverage. In Estonia and Spain in particular, long-term care is largely a family responsibility. All the countries provide informal carers with some financial support. Except for Austria and Spain, all countries support carers with flexible work arrangements. All countries provide informal carers with respite services. Additional supports, some from the voluntary sector, include informational resources and/or training, guidance and counseling, support groups, supportive technologies (Germany, Norway, Netherlands, UK) and carers’ needs assessment (UK, Netherlands).

As part of the SUSTAIN project, we conducted semi-structured interviews with informal carers of older people living at home, and receiving care and support from the integrated care initiatives participating in the project (description in Appendix-B). The different initiatives (SUSTAIN sites) included proactive primary care for older people, home nursing and rehabilitative care (both provided by healthcare professionals), transitional care, and care for people with dementia. The participating initiatives mainly centered their activities around providing and improving services for care recipients (e.g., older people) (Appendix-B). Even so, we wish to focus attention on the experiences of informal carers in these settings.

Ethical approval was provided by ethical review committees in the seven participating countries, and all respondents signed a consent form before the start of the interviews, indicating that: they agreed to participate in the SUSTAIN project, they understood the nature of their involvement, and they gave permission for interviews to be audio recorded.

### Data collection

Informal carers were recruited using convenient sampling whereby health and social care professionals invited the informal carers of the older people served by the SUSTAIN sites to participate in the study. With the informal carers’ permission, researchers scheduled and conducted interviews with informal carers (48 total: 18 older person and carer dyads, 29 carers alone). Interviews were conducted at the informal carer’s or the older person’s home, or at the SUSTAIN site. The individual, face-to-face interviews lasted approximately 60 minutes and were conducted by local researchers in each informal carer’s national/local language. The interviews explored topics related to our study aim (see the introduction) and were guided by a semi-structured interview schedule (Appendix-C: main interview questions).

Additionally, we collected data on informal carers’ sociodemographic and health service related characteristics (presented in Appendix-D). All data collection tools developed by SUSTAIN research partners were prepared in English and then translated into the integrated care initiatives’ national languages. Regular meetings and teleconferences took place between research partners to standardise the methods of data collection in each country. All interviews were audio-recorded and transcribed in the original interview language. Data about informal carers’ own experiences (as opposed to older people’s experiences) were translated into English for this study.

### Data management and analysis

Data were managed in a secure online database accessible to SUSTAIN research partners. Strict guidelines for data entry were developed and shared across research partners. Two researchers, EAA and LM, analyzed the qualitative data and cross-checked each others’ work. First, EAA read the transcribed interviews and then applied an inductive approach to identify main themes and subthemes from the content of the interviews [23] (Appendix-E) aided by NVivo 12 software. Thereafter, EAA linked key elements of person-centered care [18,19] to the identified themes (Appendix-E). LM reviewed and analyzed the coded data, and presented the findings. EAA reviewed the findings.

## Results

The findings from this study are thematically presented below. The themes identified were: assessing and responding to informal carers’ needs, understanding carers’ role over time, looking after carers’ health and wellbeing, carers’ participation in decision-making and goal-setting, and informal carers’ perceptions of how they are treated by professionals. We use female pronouns in the direct quotes keep respondents anonymous.

### Assessing and responding to informal carers’ needs

Professionals made efforts to work with informal carers in a person-centered way by paying attention to and helping informal carers’ address their needs and concerns. For example, some informal carers reported that professionals assessed their needs by asking them specific questions or, in some cases, applying standard assessment instruments. Many informal carers expressed that professionals were attentive to their needs, e.g., inquiring about carers’ wellbeing and how they were coping during home visits to the older person.

Carer: “Yes, they ask that every time they visit. ‘How are you?’ yes, they pay attention to that” (Carer1_Country1).

Professionals asked informal carers if they needed support, explained how to conduct specific care procedures, and provided useful and practical information about services or support for the carer and the older person. Some informal carers recalled talking with professionals about specific matters that interested them as carers, such as the need for respite.

Carer: “…when [older person]…was at the nursing home for rehabilitation […] I…asked [the professionals] whether they could take over the administration of her medication. And they listened to that.” […] “[Before] I controlled all her medication and picked it up at the pharmacy and ordered new prescriptions and talked with the GP… And I started to feel that it was a lot of work and also a responsibility. I talked with [older person] and she said it was OK, so I talked with home nursing and they took over” (Carer1_Country2).

However, not all informal carers reported positive person-centred experiences with professionals. Some carers expressed clearly that professionals were not attentive or responsive to their needs e.g., professionals did not ask how they were coping with the caregiving situation.

### Understanding informal carers’ role over time, and the need to balance caregiving and life/work demands

Some informal carers commented that they felt that professionals did not fully understand their situation, initially or over time. They perceived that professionals may have assumed that they would serve as caregivers by default despite their own personal challenges (e.g., physical constraints, reduced endurance/advanced age).

Carer: “I don’t think that they understand our situation. I am in much poorer health than what they believe. […] I have had two big heart attacks and two big operations. And my back is not in good shape” (Carer2_Country2).

Additionally, several informal carers complained about their own needs and schedules not being considered when professionals scheduled medical appointments for the older people. Examples of things not considered included informal carers’ need for sleep or rest, access to transport to health facilities, and the need to balance older people’s medical appointments with carers’ job demands/schedules. As such, informal carers felt they were not actively involved in decision-making about their needs, preferences, and roles as caregivers.

Carer: “Well, doctors give you what they can…, for instance, for the [older person’s technical device] they always gave her appointments in the morning, and I asked them if it could be in the afternoon, and they…[agreed]. But when it is an already scheduled visit, they say ‘that day, that time…’ and you go, …if you say you cannot go maybe they reschedule for two months later, so, best thing is not to say anything” (Carer1_Country3).

Some informal carers stressed that they had little or no follow-up communication or visits from professionals after the older person had completed rehabilitation or was discharged from hospital. Such situations left informal carers without support in their ‘new/changing’ caregiving role; and suggests that professionals performed poorly with regard to promoting cooperative relationships with carers. Informal carers also expressed that they had no follow-up from professionals after goals were set for the older person in a care plan agreed upon by the professionals, the older person and the carer. Thus, informal carers were left alone with the responsibility of supporting the older person in meeting those goals.

Carer: “They [professionals] don’t contact us to know how the situation is. And regarding the rehabilitation team that was here for 8 weeks, they have not contacted us afterwards…so it feels maybe a bit like a loss, the fact that they haven’t followed up on the work that was done here” (Carer4_Country2).

Informal carers also feared that if the older person’s health worsened such that they could not care for him/her properly, they would have to consider sharing caregiving responsibilities with others, or placing the older person in a care home. This last option was the least desired by both older people and their informal carers.

### Looking after informal carers’ health and wellbeing, including opportunities for respite

A common message that informal carers received from professionals was that ‘they should look after their own health and wellbeing too’, and not just that of the older person, in order to not get overburdened or fall ill. To this end, professionals provided carers with different types of advice, including to: eat and rest properly, go outdoors, exercise, maintain social relationships, and take breaks from caregiving. Informal carers also received psychological support or medication when needed. In these ways, professionals made efforts to be person-centred by helping address carers’ varied needs. Even so, most informal carers indicated that their health worsened after they became carers; and they expressed feeling that little could be done to change this trajectory.

Although informal carers did seek healthcare for their minor or temporary health problems, they generally tended to prioritize the needs of the older person at their own expense, despite the severity of their own health problems as carers. For example, informal carers would avoid getting surgery to address a knee problem because they felt that they could not afford to be temporarily disabled or fall ill because they believed that they should be looking after the older person. Several informal carers thus feared for what would happen when their own health gets worse, feeling that no one could take their place as carers.

Carer: “In case I have to go to a hospital…, there is no Plan B. We wouldn’t know what to do. […] My husband cannot do anything alone. He needs me very much. And [the professional] said to me that I have to look good after myself. I should not overburden myself. That’s what she said insistently to me” (Carer1_Country4).

Many informal carers were offered respite services at some point, or were encouraged to take a break from caregiving and participate in leisure activities. However, some carers declined these offers because they did not want to leave the older person ‘alone’ e.g., in a care home. Other informal carers also did not feel comfortable leaving the older person at home with a professional. This latter scenario was especially the case for older women caring for their frail or severely dependent spouses at one site, but it was also reported by men caring for their parents.

Some informal carers who tried respite services commented that it did not work well for them. For example, because of the time and effort needed to commute to an inconveniently located care home to visit the older person. However, other informal carers welcomed and appreciated the opportunity for respite.

Carer: “…it took me 2–3 hours [via public transport] to get back and forth. And then I stayed with her [the older person at the care home] for 3 hours. But it was a hassle…” (Carer4_Country2).

Carer: “I think [going to the day center] is good for her [older person], and for me as well – I have to be honest about that. It enables both of us to do a bit of something else” (Carer3_Country2).

### Informal carers’ participation in decision-making and goal-setting

Many informal carers could not remember setting any goals, together with professionals, related to their own health and wellbeing needs. Some carers anticipated that it would not be difficult being involved in such shared decision-making if/when needed. Other carers explained that they had outlined together with professionals a plan for how they would look after themselves (e.g., taking breaks, exercising, leisure activities). However, informal carers’ input about goal-setting and decision-making regarding their own wellbeing was limited and vague, overall. Informal carers (and professionals) primarily focused on older people’s needs.

Carer: “To my knowledge, there are no goals [set with the professional] … which should be achieved [by the older person or informal carer]” (Carer2_Country4).

Carer: “Yes, what kind of goals do I have? Haha, I am. Well…, what kind of goals do you still have at this age?” (Carer2_Country1).

### Informal carers’ perceptions of how they are treated by health and social care professionals

Informal carers tended to first think about how professionals treated the older person, followed by how professionals treated them as recipients of care or support. This order of priority influenced informal carers’ feelings about how they were treated by professionals, and most carers were generally pleased with the way they and the older people were treated. They described the treatment as ‘excellent’ or ‘very good’, expressing that they were treated with respect and sensitivity, and that they felt listened to.

Carer: “So, I was very satisfied, [the professional] listened very well. Really, listened very carefully” (Carer1_Country4).

Some informal carers, however, did not have positive experiences with specific professionals. These carers expressed that professionals should be sensitive and considerate of the fact that informal carers who seek their support are often vulnerable, in need, worried for their relatives, and (sometimes) stressed by caregiving demands. One informal carer described how she felt after a negative encounter thus:

Carer: “You feel like…answering rudely, but you…have to shut up because you think ‘maybe if you say something they won’t give you anything…’ [services for the older person]” (Carer2_Country3).

Specifically, with regard to communication/sharing information, informal carers were satisfied overall with the way professionals communicated with them. In particular, nurses and social workers were often regarded as ‘good’ and communicative professionals who welcomed informal carers’ questions and explained things in a clear, detailed and comprehensive way.

Carer: “When you are talking with [professionals] in person you understand it, otherwise you ask. But when you receive something [e.g., official letter …] from the government … there is jargon. […], you cannot understand it completely. After that, it is very helpful to have a professional [e.g. social worker] that tells you: ‘this is normal’” (Carer3_Country3).

Physicians, on the other hand, were described by carers as less communicative, did not have enough time to accurately explain things, or used too much jargon that made it difficult to understand the information being shared.

## Discussion

This study focused on informal carers of older people living at home, who were receiving professional care from integrated health and social care settings in Europe. It described informal carers’ experiences with caregiving and with health and social care professionals, in relation to their own needs as carers. An important aspect of integrated care is the proactive assessment of health and social care needs of the older people and their informal carers [19,24,25,26,27]. Doing so enables professionals to work in a person-centered way by understanding the specific needs and concerns of older people and their informal carers, and thereafter helping them address those needs appropriately. Findings from this study, however, show that there is room for improvement in terms of the person-centredness of the care and support that informal carers received. For instance, informal carers had mixed experiences with how attentive professionals were to their needs. Needs assessments did not always take place or were experienced as not having been performed. Additionally, across the participating countries, professionals did not always involve informal carers in setting goals and plans for addressing carers’ own health and social care needs; or in decision-making about carers’ preferences and roles. Professionals further seemed to perform poorly with regard to promoting cooperative relationships with informal carers. Moreover, although professionals made efforts to support informal carers by providing them with advice and opportunities for respite, potential barriers to carers’ uptake of support and respite (e.g., challenging emotional aspects of caregiving) were hardly discussed.

The countries represented in this study have different types of support services for informal carers that are provided by both the public and the voluntary sector (Appendix-A); and informal carers reported that professionals provided them with advice and information to support their own health and wellbeing as carers. Even so, the findings suggest that it is informal carers’ loved ones (older people) who are primarily prioritized by professionals in terms of needs assessment, goal setting and care planning. This was the case even though research indicates that the assessment of informal carers’ needs is also important [3,28]; and that informal carers living in areas where needs assessments were available had better access to information, care and support [28]. The UK and the Netherlands, two countries in the current study, are making progress in the right direction. The UK’s Care Act of 2014 [29] has a legal requirement for informal carers to have a needs assessment; and in the Netherlands, such assessments are part of the health and social care system although they are yet to be systematically and consistently implemented. Efforts such as these create room for professionals to engage informal carers around the carers’ own needs.

Existing research also shows that the establishment of an accommodating, cooperative and ongoing relationship between the professional, the person receiving care and the informal carer, including respectful communication and active listening, is an important aspect of integrated and person-centered care [30,31]. The informal carers in this study were generally satisfied with the person-centered way in which professionals interacted with them: e.g., treated them with respect, communicated with and listened to them, and took an interest in their roles as informal carers (e.g., inquired about their wellbeing and need for support). Additionally, in general across the participating countries, informal carers reported that professionals provided them with informational resources. This is consistent with informal carers’ national contexts (Appendix-A) where they have support in the form of information (e.g., knowledge, advice, and services also from voluntary organizations). Our results did however point to some areas of improvement, including that professionals should: interact with informal carers with sensitivity and consideration as carers may themselves be vulnerable (e.g., burned out, stressed), communicate with informal carers effectively (e.g., use less jargon), and follow up with carers more closely regarding the care of the older person. Taken together, these findings can provide other professionals with an understanding of what informal carers need and appreciate in their relationships with professionals. They show that professionals should continue to focus attention on person-centered care, especially developing good relationships with informal carers that create room for talking with carers and understanding their specific needs and concerns.

Another area of improvement towards better quality of care and support for informal carers, based on results from this study, is that professionals should be more attentive to how informal carers are coping with the caregiving situation, and the impact of caregiving in their daily lives. In accordance with findings from other investigators [32], several informal carers in this study indicated that there seems to be insufficient room for balancing caregiving with other aspects of their lives, including looking after their own health needs, and balancing caregiving with their work schedules and demands, family life, and leisure time. Research shows that women are overrepresented in informal caregiving [2,33]. In this study, there were twice as many female as there were male informal carers under age 65. Occupying this role has been shown to have negative consequences for women more so than men including: losses in income, career development, and social engagement linked to exiting or reducing their participation in the labor force [2,3,10]; health problems linked to the foregoing losses [2,3,33]; and health problems associated with exposure to stress emanating from the demands (and gender disparities therein) of juggling multiple social roles e.g., family, work, informal caregiving [3,16]. It is therefore essential that informal carers, and particularly women, receive good support from their families, especially their partners/spouses (e.g., equitable distribution of household responsibilities, childcare, informal caregiving); and from the state e.g., paid care leave, flexible work arrangements that facilitate balancing work and family life, childcare and opportunities for respite from caregiving—support that can reduce or protect against the negative consequences of informal caregiving.

In general, informal carers across the countries participating in this study did not report experiencing financial strain associated with their caregiving role. In Estonia and Spain, caring for family members in need of long-term care is largely the responsibility of the family. Therefore, it could be that informal carers from these countries did not expect much support from their health and social care systems, and may have underreported deficits in informal carer support services/resources. The previously mentioned gender considerations in caregiving may also be heightened for female informal carers in Estonia and Spain. Our findings on financial strain may also partly reflect informal carers’ national contexts where carers were provided with some financial support (allowance, care support benefit) and/or flexible employment arrangements (Appendix-A). The voluntary sector in many of the countries was also involved in supporting carers. Additionally, respite services (e.g., adult day centers, short-term institutional stay), and guidance/counseling and training were also available for informal carers at the national level across the participating countries. Even so, some informal carers in the study were reluctant to receive respite from caregiving because they considered themselves primarily responsible for caring for the older person, and were thus unsure about entrusting another person with the responsibility. These findings point to opportunities where professionals could have worked with informal carers in a more person-centered way. For example, through closer relationships with carers characterized by good communication, professionals could have had discussions with carers about the challenging emotional aspects of caregiving; and encouraged carers’ participation in identifying and making use of appropriate support services such as counselling.

Complex emotions are a barrier to informal carers seeking and accessing support, and they include embarrassment, guilt [1], anxieties around handing over care [34], and a desire to keep the caregiving situation private especially if the person with care needs does not want help from outside the home [1]. Caring may be seen as a normal part of the relationship between the informal carer and the care recipient, and there may also be doubts about the benefits of external support or the associated costs [35]. Open, honest and respectful relationships between informal carers and health and social care professionals are an important element of person-centered care [18,19]; and such relationships are needed to help informal carers overcome barriers to seeking and accessing support [1,3,34]. Professionals should further bear in mind that the needs of informal carers may change over time, depending on the situations of the older people they are caring for and also informal carers’ own life circumstances [3,11]. Regular assessment of informal carers needs is therefore key to delivering person-centered care characterized by professionals having a good understanding of informal carers’ specific needs and concerns, and helping carers address those specific but varied needs that they have (as whole people e.g., needs in the areas of physical and social health, complex emotions of caregiving).

The informal carers in this study tended to prioritize the health and wellbeing of their loved ones over their own needs, a finding that has also been reported in other recent studies [5,6,10,12,13]. Given the overrepresentation of women in informal caregiving [3,33], this finding may have consequences for gender disparities in the deleterious effects of caregiving. Similarly, this study and others [1,3] also show that professionals mainly focus on the needs of older people (care recipients). That informal carers’ health and wellbeing needs are secondary to those of the older person may also explain our finding that both informal carers and professionals did not proactively seek out opportunities to set goals for informal carers’ health and wellbeing, and plans for meeting them. It is very important to pay explicit attention to informal carers’ needs in order to reduce or prevent caregiver burden, reduce gender disparities in health, and maintain carers’ healthy perseverance in the caregiving role over time.

Informal carers in this study reported that professionals provided them with advice about self-care, and information about services like respite and support groups. Professionals can further help informal carers by engaging them in discussions about their needs and wishes, helping them set plans for meeting those needs, and identifying and guiding them to additional support services such as counselling, therapy, and workshops/training sessions on self-care and coping with the demands of caregiving [36,37]. That said, professionals’ busy schedules and limited experience working with informal carers on the carers’ own health and social care needs [1,3] are challenges that health and social care systems, and nations at large, must tackle if they value the contributions of informal carers; and if they are committed to providing carers with person-centered services that prioritizes carers’ needs. Findings from this study suggest that professionals made some efforts to work with informal carers in a person-centered way. However, those efforts were limited and mostly centered around treating and communicating well with informal carers, and learning about- and addressing some of their needs (e.g., informational support, advice; opportunities for respite). Other key elements of person-centered care were poorly attended to, such as promoting informal carers’ active participation in making decisions about and managing their health and social care needs, addressing carers’ varied needs (i.e., paying attention to the whole person), and providing them with coordinated care.

### Methodological considerations

A strength of this study is that it was based on qualitative data from informal carers in seven European countries, yielding findings that can lend perspective on the experiences of informal carers in Europe. The instruments used to gather data from informal carers (questionnaire for sociodemographic data, semi-structured interview guide) were jointly developed by SUSTAIN research partners in the participating countries, allowing for uniformity across the countries. The instruments were then translated into the local languages, and researchers tailored them (defining/explaining terms and concepts during the interviews) to ensure that informal carers understood the questions. We however acknowledge that differences in the professional backgrounds of SUSTAIN researchers across the seven countries, and differences in informal carers’ own backgrounds, may have influenced data collection with regard to a uniform understanding of terms, concepts and questions; and in terms of the richness of the data gathered. For example, some informal carers did not understand when asked whether their needs had been assessed and were thus not able to provide a clear answer. Even so, our thematic coding and analysis of the data allowed us to pay attention to- and consider together the similarities and differences emerging from the data and informal carers’ input.

The informal carers in this study come from different countries with different cultural backgrounds and life trajectories. These differences likely affected the way they engaged with the integrated care initiatives at each setting, and the way they responded to the different topics explored in the interviews. Even though SUSTAIN, being a cross-country project, should lend itself well to a cross-cultural analysis of informal carers’ experiences, this was not the focus of the project. We thus lack the data needed for such an analysis, which is a limitation of this study. Even so, the findings reported here do shed some needed light on informal carers’ experiences with caregiving and with health and social care professionals in Europe.

The findings of this study are based on informal carers’ own reports and perspectives, and lacks input from the older people being cared for and health and social care professionals, which is a limitation. The viewpoints of all three (older people, informal carers, professionals) would have contributed to a richer understanding of how the complex interactions between them, and the systems in which they operate (e.g., health and social care, family, work), influences how informal carers care for themselves and are cared for.

## Conclusion

The demographics of aging populations are such that an increasing number of older people are living at home with chronic health conditions requiring health and social care services. At the same time, the health and social care systems that serve older people cannot be depended upon as the only source of long-term care given budget constraints. Informal carers make substantial contributions to caregiving, however, there is a need to pay attention to- and support them in caring for their own health and wellbeing. This is important if informal carers are to continue in their role without adverse effects to themselves (e.g., burnout, ill-health, social isolation).

Findings from this study indicated that, in general, health and social care professionals listened to informal carers, treated them with respect, and made efforts to assess and respond to their needs. Even so, the results suggest the need for professionals to interact with vulnerable informal carers with sensitivity and consideration, communicate with informal carers effectively, and follow up with them more closely regarding the care of the older person. A better understanding is also needed of informal carers’ caregiving situation and how they are coping. The findings also suggest the need to proactively involve informal carers in shared decision-making aimed at identifying goals for informal carers’ health and wellbeing, and setting plans to achieve those goals. Additionally, informal carers need to be further supported in gaining a better balance between their caregiving role and their other roles, interests, and health and wellbeing needs. This includes guiding them to services that can help them address their needs, such as difficulty entrusting others with the care of the older person when needed.

## Additional File

The additional file for this article can be found as follows:

10.5334/ijic.5547.s1 Appendix. Appendices A–E.
