# Supplementary material for: A Cross-European Study of Informal Carers’ Needs in the Context of Caring for Older People, and their Experiences with Professionals Working in Integrated Care Settings
Source: Int J Integr Care. 2021 Jul 8;21(3):2. doi: 10.5334/ijic.5547 (PMC8269786; doi:10.5334/ijic.5547)
Supplement: Appendix. — Appendices A–E. [file ijic-21-3-5547-s1.pdf]

Appendix-A. Support for informal carers within the health and social care contexts of the countries participating in the SUSTAIN project

Source: Information compiled by SUSTAIN researchers

**Type of support**		**Support available at**: National/local level (X), SUSTAIN site (S)						
		Participating countries[a]						
		AT[a]	DE[a]	ES[a]	NL[a]	NO[a]	SP[a]	UK[a]
Healthcare (primary and tertiary)								
1	Universal coverage (public/taxed-based funding, private insurance, mixed public and private; out-of-pocket/co-payment for some services)	X	X	X	X	X	X	X
Long-term care and social services								
2	Universal coverage (public/taxed-based funding, private insurance, mixed public and private; out-of-pocket/co-payment for some services)		X			X		
3	Mixed (universal and means-tested coverage)	X[h]		X[g]	X		X[b,g]	X
Financial, employment, care-leave related support								
4	Financial support (allowance); Care support benefit, paid care leave	X	X	X[b]	X[b*]	X[c]	X[c](S[h])	X (S[h])
5	Employment accommodations (e.g., flexible work arrangements)		X	X	X	X		X
Support from the health and social care system								
6	Respite services and supports (e.g., provision of/referral to adult day centers, short-term institutional stay; GPS and other welfare technology)	X	X	X[b,d]	X[b,d]	X	X[d*]	X[d*]
7	Engaging voluntary sector/volunteers to support informal carers (e.g., in their role as caregivers, providing them with opportunities for respite)	S	X(S)	X[e]	X[e]	X	S[e]	X(S)
8	Information (e.g., available support services including from voluntary organizations/centers; relevant laws, carers' rights, courses)	X	X	X[b]	X	X	X	X
9	Training, guidance/counseling	X	X	X[e]	X	X	X	X
10	Supportive technology		X[e]		X	X		X
11	Informal carer's needs assessment				X[e](S)		S[e]	X(S)
Support from organizations, foundations, centers								
12	Voluntary sector engagement/support of informal carers (e.g., in their role as caregivers, practical support, opportunities for respite, advocacy for carers)	S	X[b]	X	X[b*]	X		X
13	Information (knowledge, guidance, advice; available support services; sharing of experiences with caregiving)	X	X[b]	X[e]	X	X	X[f]	X
14	Training, counseling, emotional support; guidance, advice, mentoring	X	X[b]	X[d]	X	X	X[f]	X
15	Support groups		X[b]		X	X[b]	X[f]	X[f*]
16	Supportive technology				X			X

Notes: [a]AT (Austria), DE (Germany), EE (Estonia), NL (Netherlands), NO (Norway), SP (Spain), UK (United Kingdom); [b]Service is (also[*]) organized at the local/regional level, hence there may be variation across localities;

[c]Service is selective i.e., for informal carers who meet certain criteria e.g., caregiving hours, heavy caregiving workload; [d]Service is (may also be[*]) means-tested; [e]Limited or variable/unsystematic availability; [f]Service (also[*]) provided by organizations concerned with specific diseases e.g., Alzheimers Society; [g]Caregiving/long-term care is largely considered the responsibility of the family, hence heavy dependence on informal carers; ; [h]Professionals provided carers with information about available benefits; [h]People who are not insured are not covered.

Appendix-B. Health and social care sites participating in the SUSTAIN project

Country	Integrated care site (location) & Type of services	Description of the care site & SUSTAIN improvement project objective
**Austria**	Gerontopsychiatric Centre (Vienna)<br><br>*Dementia care*	"[A] centre for community-based gerontopsychiatric consultancy (clinical-psychiatry and neurology) and serves as a service centre for patients, their families (carers) and for other stakeholders in health and social care… [The multi-professional team] … address the needs of older people suffering from cognitive decline, related morbidities and social problems."<br><br>SUSTAIN objective: To improve detection of dementia and case- and discharge management of hospitalised people identified with a cognitive disorder.
**Estonia**	Alutaguse Care Centre (Ida-Viru)<br><br>*Home nursing & rehabilitative care*	"The nursing and health care services […] are cultivated based on the real needs of senior citizens… […] The Care Centre has five different service areas: round-the-clock special-care services for adults with special mental needs; 24-hour care (nursing home service); care for persons suffering from dementia; day nursing/care; and nursing treatment (health service)."<br><br>SUSTAIN objective: To develop a person-centred way of working by engaging older people, informal caregivers and a multidisciplinary care team in the process of defining a goal-directed care plan.
	Medendi (Tallin)<br><br>*Home nursing*	"Medendi is a small private [home nursing company funded by the National Health Insurance Fund]… Home nursing aims to help service users who are recovering from operations, the elderly, the disabled and [other in need of care]. […] Its services are delivered through a multi-agency partnership with other service providers: … physiotherapists, family doctors and palliative nurses…"<br><br>SUSTAIN objective: To increase the engagement of the older person, informal caregiver and different professionals in the development of a joint care plan, and to support information exchange between the older person, informal caregivers and professionals about the older person's situation, needs and objectives.
**Germany**	KV RegioMed Zentrum Templin (Uckermark)<br>*Rehabilitative care*	"The integrated care programme of the KV RegioMed Zentrum in Templin provides a three-week complex therapy programme. During this programme service users (older people) are learning about their medication plan, falls prevention, healthy diet and lifestyles and their social needs. They receive a detailed and tailored care plan."<br><br>SUSTAIN objective: To enable people with care needs (including people who completed a complex therapy program) to receive the right services, by providing information and advice on available care and support services.
	Careworks Berlin<br><br>*Home nursing and rehabilitative care*	"The Pflegewerk Berlin (Care-Works Berlin, CWB) serves a varied population in less affluent parts of the city. … In the integrated care initiative of Pflegewerk Berlin: nurses, medicines and therapists are involved. Furthermore, the Pflegewerk [serves people] ranging from self-funded service users to those covered under social long-term care insurance and those who need support from the social services department."<br><br>SUSTAIN objective: To improve inter-professional case management and multidisciplinary collaboration between general practitioners, (para)medical therapists and nurses by transferring prescription-competence from General Practitioners to (para)medical therapists and nurses; and to establish formalised interactions and communication space among involved (formal and informal) caregivers.
**Norway**	Holistic Patient Care at Home (Surnadal)<br>*Home nursing and rehabilitative care*	"Surnadal's healthcare services are available for all residents. They include but are not limited to general practitioner (GP) services, emergency care, long-term institutional services, physiotherapy and occupational therapy, mental health and homecare services."<br><br>SUSTAIN objective: To expand and improve healthcare services delivered at home.

	Everyday Mastery Team (Søndre Nordstrand, Oslo)<br><br>*Rehabilitative care*	"Søndre Nordstrand is the largest and youngest borough in Oslo municipality. […] Everyday Mastery Training (EMT) service …provides rehabilitative care (including training in ADLs) to users in their homes for 4-8 weeks. The health and social care services provided in Søndre Nordstrand […] include emergency care, GP services, nursing homes, Day Center, senior center, homecare services, mental health services, and rehabilitation including EMT. The services are available for all residents of the borough.2<br><br>SUSTAIN objective: To increase people's sense of personal control, reduce reliance on traditional care services and maintain and encourage good functional ability and social participation among older people.
**Spain (Catalonia)**	Severe Chronic Patients/ Advanced chronic disease/ Geriatrics (Osona)<br><br>*Proactive primary and intermediate care*	"The Osona Program […in collaboration with partners] serve a population of approximately 155.000 inhabitants, of which 2.58% are users with complex health and social needs. This unique configuration brings together local public sector entities involved in the care continuum of 65+ users with chronic health conditions and complex social and health needs: primary health care nurses and GPs delivering home and ambulatory care; social workers assessing home environments and social-relational networks; specialist doctors for several chronic conditions, and acute and intermediate hospital staff caring for these users both as outpatients and inpatients."<br><br>SUSTAIN objective: To improve person-centeredness of care by conducting a standard, multidimensional joint assessment and elaborating a shared individualised care plan among involved health care and social care professionals and the older people and informal caregivers.
	Social and health care integration (Sabadell)<br><br>*Proactive primary care*	"Services [in the program] …include three Primary Health Care Centres (PHCC) in north Sabadell […]and the local social services provided by Sabadell's city council. Thirteen professionals form the small team, including: two managers (1 health and 1 social sector) and a triad of GP, nurse and social worker for each PHCC. […] The North Sabadell Social and Health Care Integration Program was launched for 65+ users with complex health and social needs. When a user met criterion for this program, they were signed-up into a shared agenda, so their case could be discussed and assessed by the triad in their monthly coordination meeting."<br><br>SUSTAIN objective: To establish a systematic, multidimensional assessment and care plan tailored to multiple health and social care needs of each older person and to establish care plans that people feel knowledgeable and active about, targeted at those unknown to social services.
**The Netherlands**	Geriatric Care Model (West-Friesland)<br><br>*Proactive primary care*	"Over the last years, [West-Friesland] has been the context for various activities targeting care and support for older people living at home with complex care needs, [… including implementation of] the Geriatric Care Model (GCM) [in one region] Municipalities in the Netherlands are legally responsible for delivering social care and support services, as well as for providing instrumental needs to help people living at home for as long as possible. They collaborate with home care organisations and social care organisations in local social community teams in order to fulfil this responsibility. Other activities in the region include comprehensive case management for people with dementia and their caregivers… Following the implementation of the GCM in West-Friesland, the regional GP organisation agreed for the West-Friesland region to be included as one of the Dutch case sites of the SUSTAIN project."<br><br>SUSTAIN objective: To improve collaboration between General Practitioners and practice nurses, case managers for people with dementia and the social community team in order for them to adequately address older people's health and social care needs; and to improve professionals' person-centred way of working.
	Good in one Go (Arnheim)<br><br>*Transitional care*	"In the Eastern part of the Netherlands, the Arnhem region…several organisations work together to deliver person-centered health and social care services for older people. 'Good in one Go'… specifically focused on crisis situations of frail older people living at home. In the project, organisations collaborated in an informal network, which means that organisations did not have any formal agreements, but aligned their activities in order to provide a comprehensive range of health and social care services in the region."<br><br>*SUSTAIN objective:* To clarify and align the various scenarios of a sudden need for more intensive care of a person living at home in a crisis (such as dementia or brain injury).
United Kingdom	Over 75 Service (Kent)<br><br>*Proactive primary care*	"Swale is the third most deprived district within Kent… In Kent, the county council is responsible for social care services and provides formal care services in residential care homes and in people's own homes. Kent County Council (KCC) manages the Kent Enablement at Home (KEaH) service …which provides up to 3 weeks' support at home for people returning from hospital. KEaH staff work with users to learn or relearn important skills they need for everyday life, such as regaining confidence following a fall. KCC also employ care navigators who help older people to stay independent in their own home. […]Swale is also served by Swale Borough Council, [which is] responsible for housing and planning, amongst other things, and provides help and advice for older people dealing with

		repairs, adaptations and home safety issues to help them to stay in their own home…"<br><br>*SUSTAIN objective:* To keep older people with long-term conditions and complex care needs at home independently for as long as possible and to improve care coordination across existing services around these people.
	Swale Home First (Kent)<br><br>*Transitional care*	"Sandgate Road Surgery is a General Practitioner (GP) Medical Centre in Folkestone in the South East of England. […] The target group for the Over 75 Service were: people aged 75 and over; frail, housebound and vulnerable; living alone or with a spouse with limited social or family support; complex health and social care needs and at high risk of hospital (re) admission. […]The Over 75 Service has a core team based at the surgery consisting of a Lead GP, Senior Nurses called 'Practice Matrons', a paramedic practitioner and administrative staff. There is a wider multidisciplinary team from health, social and voluntary sector organisations… As a result of this extensive team, a range of services are delivered including medical and nursing care, social care, health training, independence co-ordination, care navigation, carer support and medicines management."<br><br>*SUSTAIN objective:* To ensure medically optimised hospitalised people are able to be discharged straight home with the right support and to make the person's discharge smoother, quicker and safer by moving to a single assessment.
Source: Adapted from De Bruin et al., 2018 [7,35]; and SUSTAIN country reports (Austria, p. 11; Spain, p. 12, 29; Estonia p. 10, 24; UK p. 10, 25; Germany p. 10, 25; Netherlands p. 11, 26; Norway p. p. 10, 25): https://www.sustain-eu.org/products/sustain-country-reports/		

Appendix-C. Caregiver interview questions

1. How do the workers (e.g. nurses, social workers, occupational therapists, health assistants) who provide care to [older person] treat you?
    a. How do you feel about the way you are listened to and respected for what you say?
2. Do you know who [professional] to contact…if you need to ask questions about [the older person's] conditions and care?
    a. How well do you feel this person [professional] understands your…needs?
3. Do you feel the workers are meeting your own needs?
    a. Do you feel that all your own needs are assessed properly?
    b. How have your needs been assessed, and how did you experience this (e.g. did you feel safe/secure to share all the needs you have)?
4. Do you have the opportunity to discuss your needs with the health workers, and are you involved in making decisions about how your own needs (e.g., for respite) can be met?
5. Please describe any goals related to your health and wellbeing that have been set for you. How involved were you in developing the goals?
    a. Have you achieved your goals?
    b. Has a professional evaluated the extent to which you have achieved your goals?
6. To what extent are you, yourself, offered any support in dealing with any emotional effects of caring for [the older person] (e.g. support groups, respite from caring)?
7. Do you feel supported to do most of the things you want to do outside your care work? (e.g. work, socialising, physical activities, leisure, study)?

Appendix-D. Characteristics of informal carers of older people living at home (N = 44)		
Characteristic	N	Percent/ Mean(SD)
Country		
Catalonia (Spain)	5	11.4
Germany	5	11.4
Norway	8	18.2
Netherlands	12	27.3
United Kingdom	9	20.4
Austria	2	4.6
Estonia	3	6.8
Female (/male)	14	68.2
Age		
25-34 years	1	2.3
35-44	1	2.3
45-54	5	11.4
55-64	9	20.5
65-74	7	15.9
75-84	19	43.2
85+ years	2	4.5
Education		
Primary	11	25.0
Secondary	17	38.6
Further education	12	27.3
Higher education	3	6.8
Missing	1	2.3
Marital status		
Married/cohabiting	40	90.9
Separated/divorced	2	4.5
Single	2	4.5
Relationship to older person		
Spouse/partner	30	68.2
Son	5	11.4
Daughter	6	13.6
Other family/relative	2	4.5
Other	1	2.3

Living situation (proximity to older person)		
Living with the older person	29	65.5
Living close by (<5 km)	12	27.3
Living further away (>5 km)	3	6.8
Paid job		
Yes	14	31.8
No	29	65.9
Missing	1	2.3
Paid working hours per week (where reported)	13	29.4<br>(11.5)
Caregiving hours per week for carer not living with older person<br>(where reported)	13	17.0<br>(14.2)
Caregiving hours per week for all carers (where reported)	23	33.7<br>(41.6)
Carer's needs assessed by a healthcare professional		
Yes	9	20.5
No	28	63.6
Missing/don't know	7	15.9
Carer has a care plan		
Yes	4	9.1
No	28	63.6
Missing/don't know	12	27.3

Description: Table 1 shows the characteristics of 44 of the 47 informal carers in this study for whom quantitative data was available. Over two-thirds of the informal carers were female, many (63%) were age 65+, and under one-third had attained a post-secondary education. All but one informal carer were kin. Most informal carers were the spouse/partner of the older person; and sons and daughters were nearly equally represented as carers. The majority of informal carers lived with the older person. The few informal carers who had a paid job worked an average of 29 hours per week. Most informal carers who lived with an older person did not report hours spent on caregiving activities given the difficulty of disentangling them from daily household chores. Informal carers who did not live with an older person reported an average of 17 hours per week spent on caregiving (range 2-48). A minority of informal carers (20%) reported that their own needs had been assessed (16% did not know). Additionally, only one in ten informal carers report that they had their own care plan (27.3% did not know/provide the information).

Commentary: That the informal carers are somewhat homogenous (i.e., approx. 68% female, 68% spouses/partners, 63% aged 65+) suggests that women [37,40] and spouses/partners who are themselves older [37] are especially represented in informal/family care of older people. That few informal carers had higher education (6.8%) might be because many informal carers in the study are of an older generation (born in the early 1950s), a cohort who—unlike children of later decades—were less likely to attain a higher education [41].

Appendix-E. Analysis themes and subthemes

(Addressing informal carers' own health and social care needs: carers' reports of their experiences with health and social care professionals)

**Themes**	**Subthemes**	**Relevant elements of person-centered care** (also see part 2 below)
1. Assessing and responding to informal carers' needs	a. Needs assessment<br>b. Paying attention to/inquiring about carers' wellbeing, need for support<br>c. Talking with carers about matters that interests them<br>d. Providing carers with information and guidance	2. Promoting a cooperative relationship with the client (respect, active listening, good communication)<br>3. Understanding the client's specific needs and concerns (his/her preferences, priorities)<br>4. Addressing the client's varied needs (paying attention to the whole person)
2. Understanding carers' role over time, including their need to balance caregiving with life/work demands	a. Understanding carers' caregiving situation and capacity<br>b. Follow-up communication with/support for carers after the older person (care recipient) has been discharged from formal healthcare services (e.g., nursing/rehabilitation care at home)<br>c. Carers' concerns about future changes in older person's needs/situation<br>d. Taking into consideration carers' own needs and wishes when planning the older person's care	1. Promoting the client's active participation in making decisions about- and managing his/her health and social care needs<br>2. Promoting a cooperative relationship with the client (respect, active listening, good communication)<br>3. Understanding the client's specific needs and concerns (his/her preferences, priorities)<br>4. Addressing the client's varied needs (paying attention to the whole person)
3. Looking after carers' health and wellbeing; opportunities for respite	a. Advice/information for carers about taking care of their own health and wellbeing<br>b. Carers' perceptions, efforts and concerns around taking care of their health and wellbeing<br>c. Carers concerns about, and uptake of opportunities for respite	1. Promoting the client's active participation in making decisions about and managing his/her health and social care needs<br>2. Promoting a cooperative relationship with the client (respect, active listening, good communication)<br>3. Understanding the client's specific needs and concerns (his/her preferences, priorities)<br>4. Addressing the client's varied needs (paying attention to the whole person)
4. Carers' participation in decision-making and goal-setting	a. Carers setting goals regarding their health and wellbeing with professionals<br>b. Carers creating plans for meeting their health and wellbeing related goals	1. Promoting the client's active participation in making decisions about and managing his/her health and social care needs<br>3. Understanding the client's specific needs and concerns (his/her preferences, priorities)<br>4. Addressing the client's varied needs (paying attention to the whole person)
5. Carers' perceptions of how they are treated by professionals	a. How carers are met and treated by health and social care professionals<br>b. How professionals communicate with and listen to	2. Promoting a cooperative relationship with the client (respect, active listening, good communication)<br>3. Understanding the client's specific needs and concerns

	carers	(his/her preferences, priorities)<br>4. Addressing the client's varied needs (paying attention to the whole person)
**Part 2: Elements of person-centered care**		
1. Promoting the client's active participation in making decisions about and managing his/her health and social care needs<br>2. Promoting a cooperative relationship with the client (respect, active listening, good communication)<br>3. Understanding the client's specific needs and concerns (his/her preferences, priorities)<br>4. Addressing the client's varied needs (paying attention to the whole person)<br>5. Providing coordinated care		
